# Flexible and Stable GaN Piezoelectric Sensor for Motion Monitoring and Fall Warning

**DOI:** 10.3390/nano14242044

**Published:** 2024-12-20

**Authors:** Zhiling Chen, Kun Lv, Renqiang Zhao, Yaxian Lu, Ping Chen

**Affiliations:** Center On Nanoenergy Research, Guangxi Key Laboratory for Relativistic Astrophysics, School of Physical Science and Technology, Guangxi University, Nanning 530004, China

**Keywords:** piezoelectric sensor, motion monitoring, GaN nanoplates, gesture recognition, fall warning

## Abstract

Wearable devices have potential applications in health monitoring and personalized healthcare due to their portability, conformability, and excellent mechanical flexibility. However, their performance is often limited by instability in acidic or basic environments. In this study, a flexible sensor with excellent stability based on a GaN nanoplate was developed through a simple and controllable fabrication process, where the linearity and stability remained at almost 99% of the original performance for 40 days in an air atmosphere. Moreover, perfect stability was also demonstrated in acid–base environments, with pH values ranging from 1 to 13. Based on its excellent stability and piezotronic performance, a flexible device for motion monitoring was developed, capable of detecting motions such as finger, knee, and wrist bending, as well as swallowing. Furthermore, gesture recognition and intelligent fall monitoring were explored based on the bending properties. In addition, an intelligent fall warning system was proposed for the personalized healthcare application of elders by applying machine learning to analyze data collected from typical activities. Our research provides a path for stable and flexible electronics and personalized healthcare applications.

## 1. Introduction

With the rapid development of the Internet of Things (IoT), smart wearable devices are playing a crucial role in advancing human–machine interaction [1], personalized healthcare [2], and anti-counterfeiting applications [3,4]. Among them, piezoelectric sensors have been widely developed for flexible placement on different parts of the body to monitor routine physiological data such as body temperature [5], pulse [6], and breathing [7], providing comprehensive support for health monitoring, real-time health feedback, and personalized medical guidance. For example, Yang et al. [8] explored a piezoelectric generator of a ZnO nanowire to convert energy generated from finger tapping and irregular movements into electrical energy. Xue et al. [9] developed a self-powered electronic skin based on a GOx@ZnO nanowire array, responding to pressure in liquid environments. Deng et al. [10] achieved gesture recognition using ZnO homojunctions combined with machine learning, achieving an accuracy of up to 100% after training. Wang et al. [11] designed a multifunctional piezoelectric portable mask by lead zirconate titanate materials to monitor breathing frequency in real time. At the same time, wearable devices based on two-dimensional materials were exploited for their large surface area, such as molybdenum sulfide (MoS_2_) [12], tungsten diselenide (WSe_2_) [13], graphene [6], zinc sulfide (ZnS) [14], WSe_2_/MoS_2_ [15], and so on, along with various applications in the humidity sensor [16], electronic skin [17], irregular breathing detection [18], human-machine interfaces [19], gas sensing [20], environmental monitoring [21], and others. However, the long-term stability of the materials was rarely mentioned for the redox reaction in air.

In recent years, gallium nitride (GaN) materials have shown great application potential in personalized healthcare due to their high sensitivity and environmental adaptability. GaN films were first developed for wearable devices. A piezoelectric pulse sensor was fabricated to accurately capture subtle waveform differences at various arterial sites, providing new avenues for monitoring physical health and assessing blood pressure [22]. Then, an eye movement sensor using GaN films was produced to detect movements of the eyeball and eyelids [23], such as blink frequency, blink duration, eye fatigue, tiredness, and drowsiness. A fully integrated and flexible patch for wireless intelligent respiratory monitoring was developed using porous GaN films [24]. This wireless and flexible patch was attached to a mask and successfully monitored seven different breathing patterns: normal breathing, rapid breathing, deep breathing, coughing, nasal congestion, sneezing, and apnea. The development of monitoring for subtle vibrations and a smart system indicates the great potential and advantages of GaN films in wearable devices and personalized healthcare. However, the films limit the resolution of flexible devices, promoting the building of smart sensors based on GaN nanorods [25,26,27,28]. A core–shell structure of GaN/CoNiO_x_ was designed and constructed based on a p-n GaN homojunction [29]. Glucose was detected using a photoelectrochemical reaction at the nanowire/electrolyte interface. The accuracy of detecting glucose in diluted blood electrolyte solutions was comparable to hospital testing values. However, the sensor suffered from a decline in performance after 27.5 h of repeated switching tests. Although GaN holds great potential in wearable devices (Table S1), the stability of GaN sensors has been overlooked, where temperature [30], humidity [31], and pH [32] can induce the changes in surface charge distribution and material properties, thereby compromising the stability of the sensors. Thus, further exploration of GaN is needed to develop sensors that leverage its piezoelectric sensitivity and resistance to harsh environments, while ensuring they are convenient to wear and functional in complex settings.

At the same time, it is reported that approximately one in four older adults in the United States falls each year, which can significantly harm their health [33]. Older adults with conditions such as osteoporosis, hearing loss, Parkinson’s disease, or central obesity are at a higher risk of falling, as these conditions can lead to decreased balance or coordination. Immediate medical intervention after a fall is critical to minimize complications such as internal bleeding, infection, and reduced mobility due to fractures or head injuries. The timely detection and treatment of falls in older adults are essential for improving health outcomes and preventing further complications.

Herein, flexible GaN sensors were prepared to improve the piezoelectric sensitivity and resistance to harsh environments, where GaN nanoplates were chosen as the materials for the lower specific surface area compared with the nanorods and higher flexibility than films. The linear piezoelectric properties of flexible GaN sensors have been applied to monitor subtle movements such as swallowing, walking, finger bending, and wrist bending. Based on the sensitivity and stability of GaN nanoplates, gesture recognition and a smart fall-warning system for the elderly was developed using machine learning (ML) algorithms to analyze output waveforms. The sensor based on GaN nanoplates combines portability, flexibility, and sensitivity to subtle changes, highlighting its significant potential in personalized healthcare monitoring for people with hearing or speech impairments and elderlypeople.

## 2. Materials and Methods

### 2.1. Materials

Tungsten (W) foil (purity 99.95%) and gallium (Ga) pellets (6 mm diameter, purity 99.9999%) were purchased from Alfa Aesar China (Tianjin, China) Co., Ltd. Urea (purity 99.999%) was purchased from Shanghai Aladdin Biochemical Technology China Co., Ltd.

### 2.2. Synthesis of GaN Nanoplates Using the CVD Method

GaN nanoplates were synthesized using the CVD method [34,35,36]. First, the W foil was treated with acetone, alcohol, and ultrapure water through ultrasonication, followed by drying with N_2_ gas. Ga pellets were placed in hot ethanol, dispersed into small droplets, and then frozen to form small Ga balls (about 1 mg). Urea was placed in a quartz boat and pretreated for 5 min in a tubular furnace under a 100 sccm 10%H_2_/90%Ar gas at 180 °C. Then, the Ga balls were positioned on the surface of the W foil and placed in the center reaction zone of the tubular furnace. The quartz boat containing the pretreated urea (about 10 mg) was placed upstream in the furnace. The furnace was purged under a 200 sccm Ar gas flow for 5–10 min to remove air. The Ga-tungsten (Ga-W) substrate was heated to 1080 °C at a rate of 30 °C/min under a 300 sccm 10%H_2_/90%Ar gas flow. Once the target temperature was reached, the urea was moved to a region heated to 180 °C by a heating belt, and the timer was set for 5 min. After the growth was completed, the system was cooled to room temperature under H_2_/Ar gas flow.

### 2.3. Fabrication of GaN Sensor

To fabricate GaN devices, the GaN nanoplates need to be transferred onto PET using PMMA [13,37,38]. Poly (methyl methacrylate) (PMMA) was spin-coated onto the GaN growth substrate and heated at 140 °C for 5 min. The PMMA around the PMMA/sample substrate was scraped off using tweezers and etched in hydrochloric acid for 2 h to obtain the PMMA/sample piece. Then, PMMA was washed with ultrapure water several times and transferred to the PET substrate. Finally, the PET substrate was soaked in acetone at 60 °C to remove the PMMA. After being dried by N_2_ gas, Ag electrodes were deposited on the GaN/PET to fabricate the sensor.

### 2.4. Characterization

Scanning electron microscopy analysis was performed on a field emission scanning electron microscope (ZEISS, Sigma500, Cambridge, UK). The X-ray diffraction (XRD) pattern of GaN nanoplates was obtained on a Malvern Panalytical X’PERT3 MRD diffractometer (Malvern Panalytical, Beijing, China) with a slit of 0.02° at a scanning rate of 5° min^−1^ using Cu K_α_ radiation (λ = 1.5406 Å). Raman spectroscopy was performed on a spectrometer (CNIlaser, U-532-100mW-19041793, Changchun, China) equipped with a 532 nm laser. Photoluminescence (PL) spectroscopy was performed on a spectrometer (CryLas, FQCW266-50, Berlin, Germany) equipped with a 266 nm laser. The XRD pattern, Raman spectroscopy, and PL spectroscopy were performed with the GaN nanoplates transferred onto a Si substrate.

### 2.5. Measurement

The GaN sensor was fixed on a custom-made holder attached to a linear motor (NTI AG, B01-37x166, Lake Geneva, WI, USA), which controls the speed, acceleration, and amplitude of the sensor. An electrometer (Keithley, 6514, Shanghai, China) was used to collect the output voltage from the sensor. Humidity-related tests were conducted inside a glove box (TONGRUN, AGB-4T, Xiamen, China) measuring 50 cm × 50 cm × 90 cm. The ambient temperature was controlled by a temperature and humidity control system (DST, RA-51R, Nanjing, China). For temperature-related tests, a temperature controller heating system (MEIKONG, TN99D-15A, Taizhou, China) was used in conjunction with an infrared thermometer (MESTEI, IR03A, Shenzhen, China) to regulate the temperature. Acidic solutions with pH values ranging from 1 to 6 were prepared using concentrated hydrochloric acid (KESHI, AR, Chengdu, China) and deionized water, while alkaline solutions with pH values from 8 to 13 were prepared using sodium hydroxide (KESHI, AR) and deionized water. The pH values were determined using a pH meter (LICHEN, PH-100, Shanghai, China).

### 2.6. Machine Learning Training

The machine learning code was programmed in Python v. 3.13.0, utilizing a random forest model to predict fall detection. In the data processing stage, the system extracted acceleration and angular velocity as feature values and applied standardization to the data. The standardized data served as inputs for training the random forest classifier, allowing the model to learn and recognize the distribution patterns of normal and abnormal states. Once training was completed, the model could be used to predict whether new data correspond to an abnormal state.

### 2.7. Smart Fall Monitoring System

Data collection was performed using an electrometer (Keithley, 6514) to record data at a frequency of 1000 Hz, which was transmitted in real-time to a computer. The data were then input into the pre-trained random forest model. Finally, the results were sent via a local area network to the caregiver’s mobile phone for display and alert notifications. To evaluate the feasibility of the GaN sensor for fall detection, a smart fall warning system was designed, leveraging the sensor’s high sensitivity to human motion signals. Motion data from various states, including walking, running, standing, and falling, were collected with the sensor attached to the wearer’s ankle. Anomalous waveforms associated with fall events were extracted to build an anomaly dataset for machine learning training. A random forest model, programmed in Python, was employed for classification. Features such as acceleration and angular velocity were extracted from the data and standardized before being input into the model. This approach allowed the system to differentiate between normal and abnormal motion states with improved accuracy.

## 3. Result and Discussion

**Figure 1:**
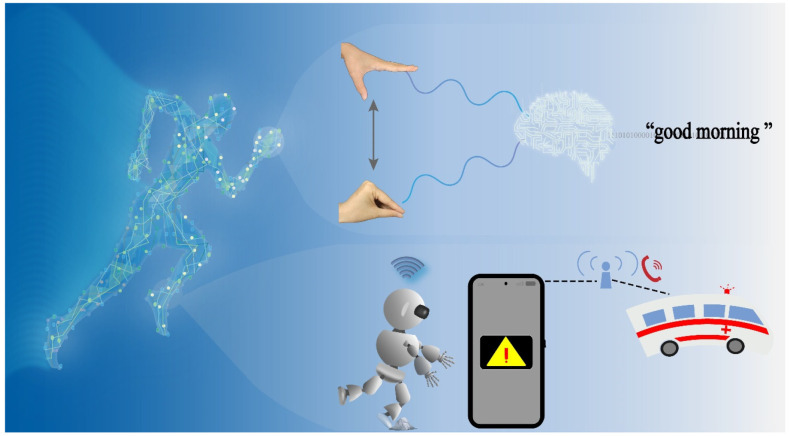
Applications of GaN sensors for motion monitoring and fall detection.

Golden hour rescue for elders when falling is the most vital part of surgical intervention for reducing multiple concurrent diseases [39,40], highlighting the urgent need for wearable and remote monitoring sensors. To address this concern, we developed a motion monitoring and warning system based on stable GaN nanoplates (Figure 1). The wearable sensor detected output signals corresponding to joint movements, enabling the inference of physical activity in the body. For instance, by sensing the output signal from finger gestures, sign language expressions were reconstructed, and gesture recognition was achieved. When sensing the signal from the ankle, walking, running, and falling were monitored and recognized. In particular, the smart warning system for elder fall detection analyzed the output waveform during a fall and triggered a warning automatically. The warning message was sent to a mobile phone spontaneously to call for help, maximizing the effectiveness of golden hour rescue efforts and reducing multiple concurrent diseases.

**Figure 2:**
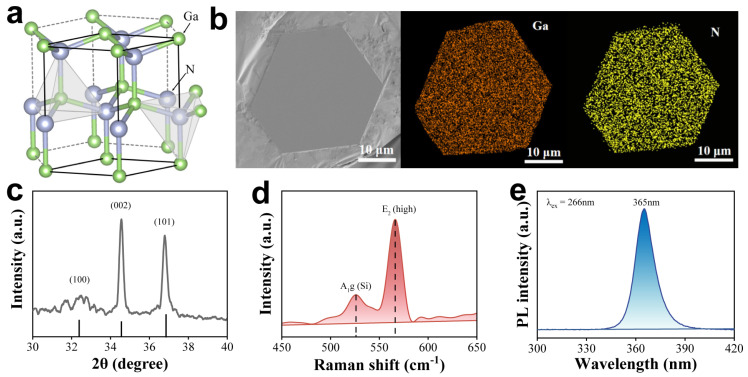
Structure and characterization of GaN nanoplates. (**a**) Wurtzite structure of GaN. (**b**) SEM and EDS mapping images of GaN nanoplate. (**c**) XRD image of GaN. (**d**,**e**) Raman (**d**) and PL (**e**) spectra of GaN, excited by 532 nm and 266 nm, respectively.

GaN nanoplates were chosen as the material for sensing subtle vibrations due to their inherent non-centrosymmetric structure and electrical power generation characteristics (Figure 2a) [23,41]. GaN nanoplates were synthesized on a tungsten substrate via chemical vapor deposition by carefully controlling the H_2_ concentration. A scanning electron microscopy (SEM) image of the nanoplate showed that the size was as large as 30 μm (Figure 2b). Energy-dispersive spectroscopy (EDS) mapping of the nanoplate showed that the element ratio of Ga and N was almost 1:1, accompanying with the uniform distribution of Ga and N elements. The X-ray diffraction (XRD) spectrum of nanoplates was aligned well with a wurtzite structure (Figure 2c), indicating the hexagonal wurtzite structure of GaN nanoplates [42], suggesting the piezoelectric potential of GaN for electrical power generation through vibration. When excited by a 532 nm laser at room temperature, the micro-Raman spectroscopy of the GaN nanoplate revealed a prominent peak at 566 cm^−1^, which corresponds to the E_2_ (high) phonon mode of GaN, confirming its wurtzite structure again (Figure 2d). Additionally, a peak at 522 cm^−1^ was also observed, attributed to the silicon substrate [43,44]. Moreover, the in situ photoluminescence (PL) spectrum of GaN exhibited a prominent ultraviolet emission peak around 365 nm, when excited by a 266 nm laser at room temperature (Figure 2e), which aligns with the peak positions reported previously [45,46].

**Figure 3:**
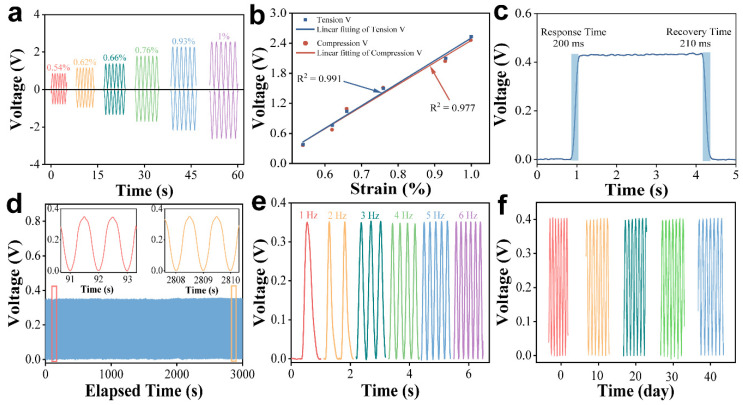
Piezoelectric performance of the sensor based on GaN nanoplates. (**a**) Output voltage of GaN sensors with different external strain. (**b**) Dependence of output voltage and external strain. (**c**) The response/recovery time of the GaN sensor. (**d**) Durability test of sensor for 3000 tensile-recovery cycles with a strain of 0.54% in 1 Hz. Insets are the output voltage of sensor at different times. (**e**) Output voltage at different frequencies under a strain of 0.54%. (**f**) Output voltage stability under 40 days, measured under 0.54% strain.

To evaluate the performance of GaN nanoplate, it was transferred onto a flexible polyethylene terephthalate (PET) substrate, and silver electrodes were deposited to fabricate the sensor. Considering tensile and compressive bending strains of various body joints, output voltage of the GaN sensor was tested under both tensile and compressive strains, ranging from 0.54% to 1%, as shown in Figure 3a. The strain was calculated based on bending conditions and is provided in Supporting Information (Figure S1) [47]. The sensor’s output voltage was generated due to the piezoelectric effect of GaN (Figure 3a) [35]. The output voltage rose from 0.37 V to 2.50 V as the tensile and compressive strains increased. The reversed output voltages for tensile and compressive stress correspond to the opposing electric fields generated by these stress states [23]. The linearity (R^2^) of the sensor was fitted and calculated to be 0.977 under a compressive strain (Figure 3b), which demonstrates a higher linearity compared to certain composite materials [48,49]. The perfect linear performance suggested the good response of smart and flexible devices. Furthermore, response speed is a critical parameter for the timely detection capabilities of piezoelectric sensors. To evaluate the response and recovery times, the output potential of the device was measured during a single strain cycle. The sensor demonstrated a response time of 200 ms and a recovery time of 210 ms (Figure 3c), enabling effective detection of subtle signals in real-life applications, which are faster than lots of hydrogel-based flexible sensors and require a quick response for body joints [50,51].

**Figure 4:**
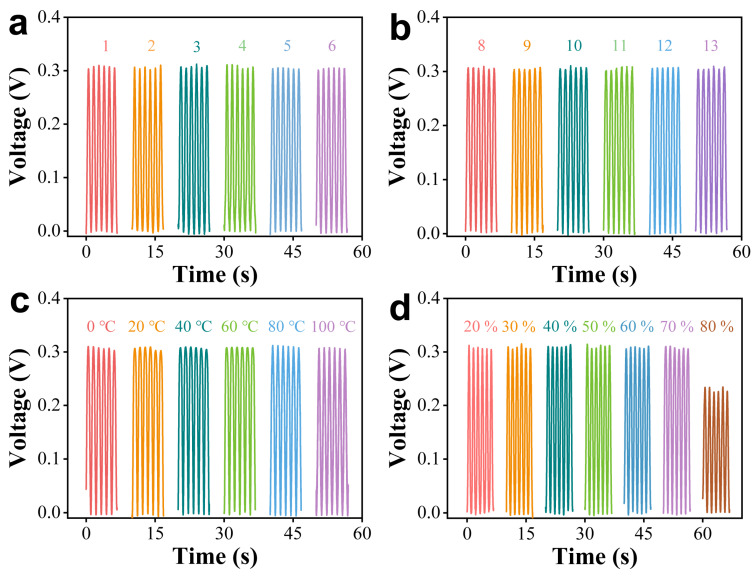
Output voltage of GaN sensors under various conditions: (**a**) pH from 1 to 6, (**b**) pH from 8 to 13, (**c**) temperature from 0 °C to 100 °C, and (**d**) relative humidity from 20% to 80%.

Stability is another vital factor for smart and flexible devices. First, a durability test was performed on the GaN sensor. The output voltage of the sensor maintained its original performance when it underwent 3000 cycles of tensile recovery testing under a strain of 0.54% (Figure 3d). This phenomenon indicated that the sensor demonstrates consistent and reliable performance over extensive strain cycling. Second, output voltage of sensor under different frequencies was evaluated to assess the possibility of sensing different human movements. Unlike other reports of frequency-dependent GaN output [35], our sensor’s voltage value exhibited good stability with minimal variation under a tensile strain of 0.54% and frequencies ranging from 1 Hz to 6 Hz (Figure 3e). This performance suggested that the sensor can reliably capture subtle changes in human motion signals. Third, to evaluate the long-term usability of the sensor, the output voltage of the same device was tested for 10 days under a strain of 0.54% at room temperature (Figure 3f). The voltage values remained stable at the initial state after 10, 20, 30, and 40 days, revealing consistent performance of GaN flexible devices in ambient conditions. Finally, considering the potential environmental condition of the human body, the output stability was tested under various conditions, including temperature, humidity (RH), acidity, and alkalinity (Figure 4). Although the output decreased from 0.35 V to 0.28 V at 80% RH, the voltage remained nearly unchanged across a pH range from 1 to 13, temperature range from 0 °C to 100 °C, and relative humidity from 20% to 70%. Additionally, a polarity switching test was performed to evaluate the consistency of GaN flexible devices under varying electrical conditions, confirming their stable operation during polarity reversals (Figure S2). The excellent stability under harsh environment reveals the huge potential of GaN sensor for smart and flexible devices.

**Figure 5:**
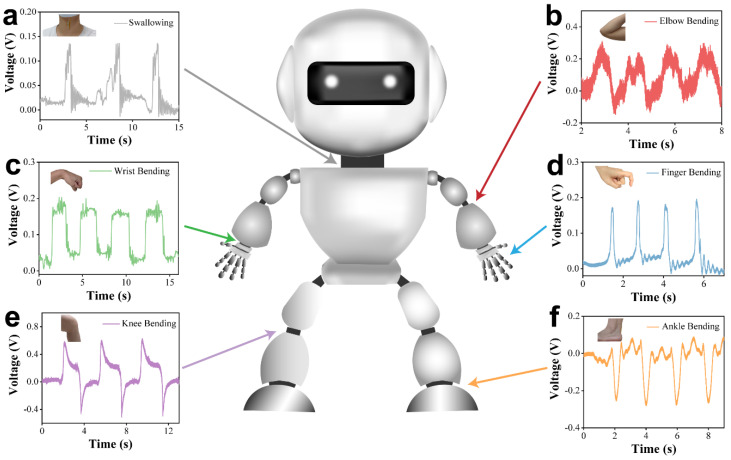
Motion monitoring of GaN flexible sensor. The response of sensor to various motions: (**a**) swallowing, (**b**) elbow bending, (**c**) wrist bending, (**d**) finger bending, (**e**) knee bending, and (**f**) ankle bending.

The exceptional and stable electrical performance indicates the capability of detecting the motion of human joints. The flexible sensor was placed on various joints to monitor human activity. It was positioned on the throat (Figure 5a), elbow (Figure 5b), wrist (Figure 5c), finger (Figure 5d), knee (Figure 5e), and ankle (Figure 5f) to monitor the bending motions (Video S1). The sensor effectively captured physiological signals, from subtle to substantial variations, with consistent voltage recorded across different areas of human body. These results indicate that the application of the sensor to the human body is feasible.

**Figure 6:**
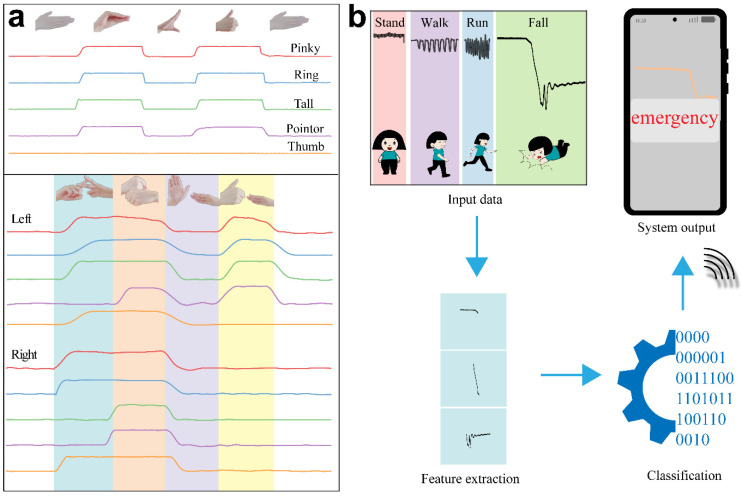
(**a**) The response of GaN flexible sensor on different fingers to sign language “Good morning” and “Wishing you success at work”. (**b**) Smart system of fall warning for elders based on GaN sensors.

Furthermore, the linear output of GaN flexible devices enables the sensor to function as a communication aid for individuals with hearing impairments, effectively conveying information through sign language. Sign language can be articulated using one hand or both hands to form gestures, which represent longer phrases. Thus, ten sensors were affixed to the joints of a participant’s fingers with sticky tape. Following the Chinese sign language system, gestures for “Good morning” and “Wishing you success at work” were performed, resulting in corresponding outputs from each finger, as illustrated in Figure 6a. The output voltages can clearly distinguish between the single gesture for “Good morning” and the two-handed gesture for the longer phrase “Wishing you success at work”. By integrating the output responses from all fingers, gesture recognition can be achieved by extracting and interpreting the intended meaning.

A smart system of fall warning for elders was developed based on the high sensitivity of the GaN sensor in capturing human motion signals. To preliminarily evaluate the feasibility of the sensor for fall detection, we designed a system in which the sensor was attached to the wearer’s ankle to collect data with various motions, including walking, running, standing, and falling events (Figure 6b). The output feature of fall events was extracted as anomalous waveforms to build an anomaly dataset for model training. Then, the collected data with various states could be classified to be normal and anomalous data by machine learning, where anomalous data are the falling events of the elderly. The machine learning approach was employed for falling detection. Considering the necessitating substantial resources of a neural network and careful parameter tuning of support vector machine, a random forest model composed of multiple individual decision trees was chosen. This model aggregates decisions from multiple trees to limit overfitting and reduce errors [52], aiming to enhance the accuracy of anomaly detection. During actual system operation, the sensor continuously monitors the wearer’s movement status in real time, and the results are synchronized to the monitor’s mobile phone. If an abnormal event is detected, a warning alert will appear on the monitor’s mobile device wirelessly to remind an immediate action to assist the fallen elders, as shown in Video S2. The falling warning of elders in real time and immediate rescue will largely reduce the delay time, increase the golden hour rescue, and lessen multiple concurrent diseases.

## 4. Conclusions

In summary, a flexible and excellent stability sensor based on wurtzite GaN nanoplate was produced, where the sensor almost kept the original performance with pH from 1 to 13, temperature from 0 °C to 100 °C, relative humidity from 20% to 70%, frequencies from 1 Hz to 6 Hz, durability test of 3000 tensile-recovery cycles, and 40 days in the air. The output voltages of GaN sensor exhibited a linearity (R^2^) of 0.977 under compressive strain, enabling precise detection of subtle vibrations in body joints, such as finger, knee, and wrist bending, as well as swallowing. Moreover, the sign language phrases “Good morning” and “Wishing you success at work” were distinguished based on the output voltages from the fingers. In addition, a smart and wearable system of fall warning for elders was designed and fabricated. Real-time monitoring and machine learning were applied based on a random forest model composed of multiple individual decision trees. This smart system accurately monitors human motion and rapidly sends alert notifications to a mobile device upon detecting a fall event, allowing caregivers to respond promptly and ensure the safety of elderly individuals. Our research may provide a pathway for real-time monitoring of elder falls, helping to increase the number of golden hour rescues.

## Data Availability

Data are contained within the article.

## Supplementary Materials

The following supporting information can be downloaded at: https://www.mdpi.com/article/10.3390/nano14242044/s1, Table S1. Summary of Applications of Wearable Devices Based on GaN Materials. Figure S1. Schematic diagram of tensile bending and compressive bending. Figure S2. “Polarity switching” test of the GaN sensor. Dynamic monitoring of human motion (swallowing, finger bending, elbow bending, wrist bending, knee bending, ankle bending) (Video S1) (MP4). Dynamic smart warning system for monitoring fall accidents (Video S2) (MP4). Refs. [35,47,53,54,55,56,57,58,59,60,61,62] are cited in the Supplementary Materials.
